# The Role of Gene Therapy as an Emerging Treatment Strategy for Alpha-1 Antitrypsin Deficiency-Associated Lung Disease: A Systematic Review

**DOI:** 10.7759/cureus.79286

**Published:** 2025-02-19

**Authors:** Shanta Afrin, Tahira Binte Hasan, MD Taraque Aziz Sagar, Tasnim Naima, Sadia Maisha, Shamima Yeasmin, Abdullah Al Masud, Fahima Akter

**Affiliations:** 1 Internal Medicine, Dhaka Medical College Hospital, Dhaka, BGD; 2 Internal Medicine, SSM Health St Mary’s Hospital, St Louis, USA; 3 Internal Medicine, East West Medical College and Hospital, Dhaka, BGD; 4 Internal Medicine, BGC Trust Medical College, Chittagong, BGD; 5 Internal Medicine, Sher-E-Bangla Medical College, Barisal, BGD; 6 Obstetrics and Gynecology, Impulse Hospital Limited, Dhaka, BGD; 7 Internal Medicine, Saint Louis University School of Medicine, Saint Louis, USA; 8 Administration, Dream USMLE, St louis, USA; 9 Internal Medicine, Indiana University Health Ball Memorial Hospital, Indiana, USA

**Keywords:** alpha 1 anti-trypsin deficiency, alpha-1 antitrypsin deficiency (aatd), chronic lung disease, gene editing, gene-replacement therapy, genetic analysis, pulmonary disease, respiratory tract infections, systematic review and meta-analysis, therapeutic interventions

## Abstract

Monogenetic disease alpha-1 antitrypsin deficiency (AATD) is the leading cause of emphysema, which is a major life-limiting chronic obstructive pulmonary disease (COPD). The current standard of care for severely affected individuals with lung disease is the periodic intravenous infusion of human AAT protein to restore circulating AAT levels to a protective level, known as augmentation therapy.

We did a systematic review to see the effect of gene therapy as a potential therapeutic option for AATD-related lung diseases. MEDLINE (via PubMed), SCOPUS, Web of Science, Cochrane Library, and EMBASE have been searched following PICO (Population, Intervention, Comparison, Outcome) criteria. After duplication removal, abstract and title screening, full-text screening was done by two individual reviewers. Then, the data were extracted, tabulated, and analyzed. A total of 1094 articles were found in the primary search. After a comprehensive review following strict inclusion and exclusion criteria, 14 articles have been included in the review.

Evidence shows the response of gene therapy depends on multiple factors, e.g., what vector is used, route of therapy administration, duration of therapy, etc. The AAV8-CASI-luc vector, delivered intratracheally (IT), achieved sustained lung transgene expression for at least 52 weeks, but 29% of mice had persistent expression up to 72 weeks, providing therapeutic AAT protein levels, reducing experimental emphysema severity in mice. Intratracheal AAV8 in mice showed the highest AAT expression in the lung, outperforming AAV9, AAV5, AAV2, and AAV2 capsid mutants, providing long-term expression up to 4 months. Intrapleural administration of AAV5-hA1AT achieved higher lung and serum A1AT levels than intramuscular delivery, with AAV5 yielding 10 times higher levels than AAV2.

Gene therapy using viral vectors has a potential role in producing AAT protein, which can be beneficial for AATD-related lung diseases. Human trials are necessary to establish the effectiveness and safety of gene therapy. In conclusion, while initial studies are encouraging, more research is needed to confirm the role of gene therapy.

## Introduction and background

Alfa-1-antitrypsin (AAT) is a protein produced in liver functions to protect the lungs from damage caused by neutrophil elastase (NE). This enzyme breaks down a crucial component of lung tissues named elastin. AAT inhibits NE and maintains the integrity of the lungs [1]. In AAT deficiency, alveoli in the lungs get destroyed, leading to difficulty breathing and gas exchange, known as emphysema, which eventually leads to a major life-limiting condition known as chronic obstructive pulmonary disease (COPD) [2]. 

The current standard of care for severely affected individuals with lung disease is the periodic intravenous infusion of human AAT protein to restore circulating AAT levels to a protective level; this is known as augmentation therapy [3]. 

Intravenous alpha-1 antitrypsin (IV-AAT) shows a significant and demonstrable survival advantage in people with severe AAT deficiency (AATD), but there is a disconnect between survival and spirometric lung function in AATD. Most randomized controlled trials of IV-AAT to date have been unable to show improvement in forced expiratory volume in one second (FEV1) after getting IV-AAT. However, the studies included participants whose lung functions had reached a low FEV1 plateau, mitigating against showing a significant therapeutic effect of IV-AAT [4]. Moreover, there is limited powerful research to demonstrate if augmentation therapy is helpful for the non-emphysema phenotypes of AATD [5]. Also, since AAT augmentation therapy requires weekly to monthly intravenous infusion of AAT purified from pooled human plasma, it has the risk of viral contamination and allergic reactions and is costly. As an alternative, gene therapy offers the advantage of a single administration, eliminating the burden of protein infusion and reducing risks and costs [1]. 

This review aims to explore the diverse strategies for AAT gene therapy targeting the pulmonary manifestations of AAT deficiency, as well as the current advancements in translating AAT gene therapy into clinical practice.

## Review

Methods

This systematic review was registered in OSF (Open Science Framework) and carried out under the Preferred Reporting Items for Systematic Reviews and Meta-Analysis (PRISMA) criteria in 2020.

Eligibility Criteria

For the systematic review, the role of gene therapy as a cutting-edge therapeutic approach for lung disease linked to alpha-1-antitrypsin (AAT) deficiency was considered. There was no restriction on the published year. We included studies related to lung disease due to AAT deficiency. We excluded case reports, case series, review articles, editorials, comments, opinion papers, letters to the editor, conference abstracts, book reports, etc. Table 1 lists the study's inclusion and exclusion criteria. 

**Table 1:** Inclusion and Exclusion criteria of this systematic review. AAT: alpha-1 antitrypsin

Inclusion criteria	Exclusion criteria
1. All study designs, including randomized controlled trials, non-randomized trials, analytic studies, and observational studies will be considered.	1. Other causes of emphysematous lung disease, except the ones caused due to AAT deficiency.
2. People with lung disease due to alpha-1 antitrypsin (AAT) deficiency, irrespective of age, gender, ethnicity, and geographic location, as well as animal studies will be included.	2. Other treatment modalities of AAT deficiency, excluding gene therapy.
3. Studies that include both AAT deficiency-associated lung disease and gene therapy will be included.	3. Case reports, case series, review articles, editorials, comments, opinion papers, letters to the editor, conference abstracts, book reports, etc.
4. Only articles written in English and published studies will be included.	4. Studies published in languages other than English.

Search Methodology

Several databases, including MEDLINE (via PubMed), SCOPUS, Web of Science, Cochrane Library, and EMBASE, were searched using a thorough search approach. Using the PICO criteria (Population, Intervention, Comparison, Outcome), we devised a thorough search strategy and used the keywords "alpha one antitrypsin deficiency" and "gene therapy" to carry out our search. These were applied singly or in combination. This systematic review focuses on gene therapy as an emerging treatment strategy for alpha-1 antitrypsin deficiency (AATD)- associated lung disease. The population includes both humans and animals, and gene therapy is the intervention. As this is a systematic review, no direct comparison is included. The primary outcome is to evaluate the therapeutic potential and effectiveness of gene therapy in managing AATD-related lung disease. The entire database search took place in March 2023 and was finished in one week. 

Screening Process

Upon concluding the database search, we eliminated all duplicate entries using Rayyan QCRI software. For the remaining articles, two independent reviewers screened the titles and abstracts. A third reviewer arbitrated any disagreements that arose between the two reviewers. Studies that weren't relevant were eliminated, and papers that looked appropriate were downloaded for full-text screening. Based on our inclusion criteria, four reviewers working in two-person teams screened each downloaded article for content in its entirety. There were stated reasons for exclusion. The two teams collaborated to address any disagreements about the inclusion, consulting the senior author for expert input.

Data Extraction

We searched every article for pertinent information, noting it in the data extraction form. Two independent review writers extracted the data, which included the study's design, year of study, sample size, gene therapy participants, placebo participants, what kind of vector used for gene therapy, dose, duration of therapy, route of administration, P value, outcome stratification, outcomes subgroups, time for immune response, side effects after administration, study limitations. Every reviewer of this study participated in a group discussion to settle any differences in the gathered data. 

Data Synthesis

To investigate the relationships within the data, compare and contrast the research, and gauge the quality of the evidence, we conducted a narrative data synthesis. Because of the heterogeneity of the included publications, a meta-analysis of this systematic review was not feasible. Nonetheless, the studies that were included varied widely in terms of the age range of the population, the study design, and the outcome assessment.

Results

Characteristics of Included Studies

In the first search, a total of 1094 articles that matched our criteria were found in the databases (MEDLINE through PubMed = 204 articles, Web of Science = 126 articles, SCOPUS = 690 articles, Cochrane Library = 5 articles, and EMBASE = 69 articles). Three hundred thirty-seven of these were eliminated as duplicates. For the remaining 757 papers, title and abstract screening was completed. To conduct a full-text screening, 110 papers were subsequently downloaded. Following the inclusion and exclusion criteria, a thorough assessment resulted in the exclusion of 96 articles in total. The following are the reasons for exclusion: Other causes of emphysematous lung disease, except the ones caused due to alpha one antitrypsin deficiency (n = 13); other treatment modalities of alpha one antitrypsin deficiency excluding gene therapy (n = 2); Case reports, case series, review articles, editorials, comments, opinion paper, letter to the editor, conference abstracts, book report etc. (n = 60); studies published in languages other than English (n = 6); full text not available (n = 15). Finally, data extraction was performed on 14 publications.

**Figure 1:**
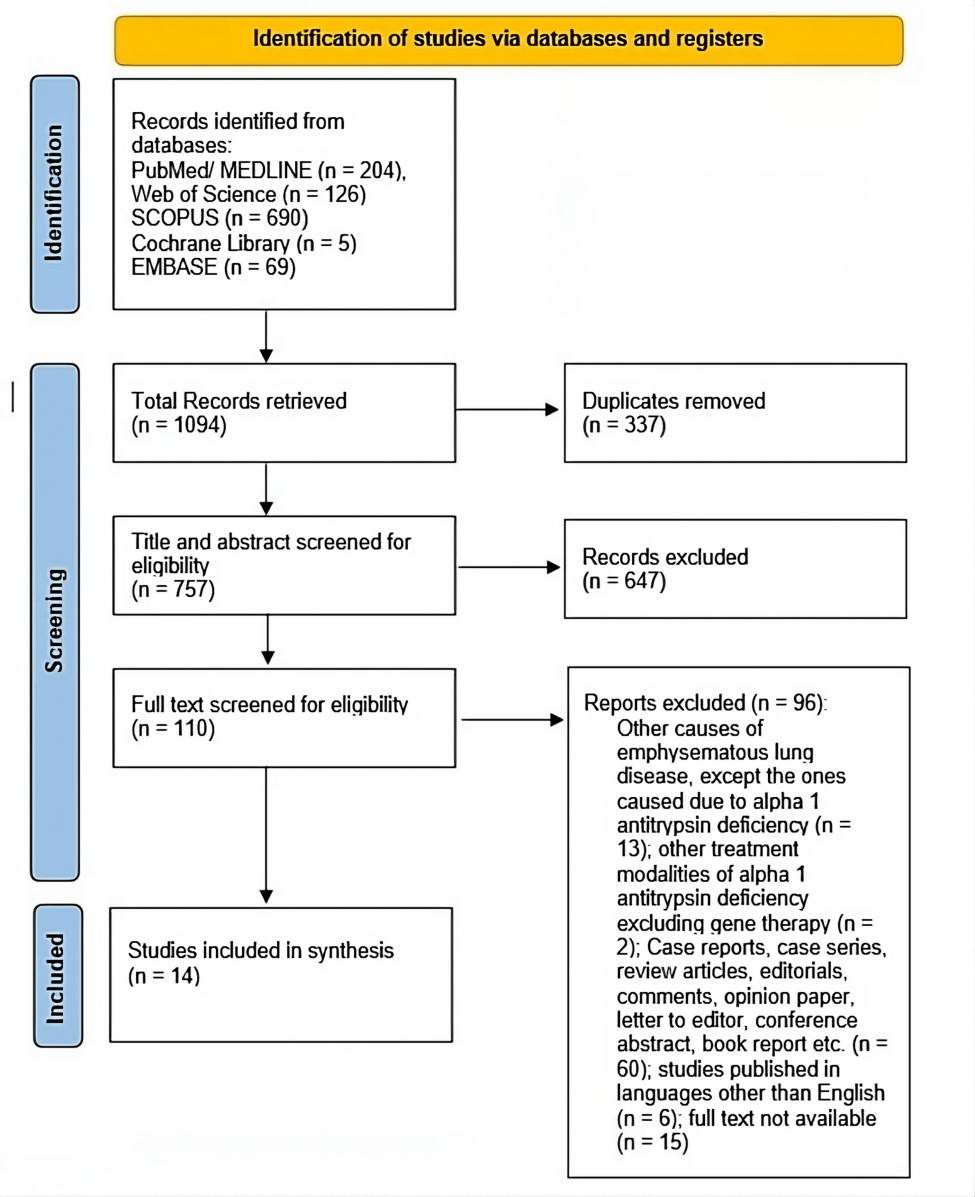
The PRISMA flow diagram. PRISMA: Preferred Reporting Items for Systematic Reviews and Meta-Analyses

The PRISMA flow diagram (Figure 1), which applies the customized inclusion and exclusion criteria, illustrates how studies were found and included in this systematic review. Table 2 summarizes the study results and baseline characteristics of the fourteen included papers.

**Table 2:** Summary of study results and baseline characteristics of the 14 included papers.

Author	Study period	Study design	Publication year	Area of study	WHO region	Gender
Marina Zieger [2]	36 weeks	Preclinical study	2022	USA	Region of Americas.	Animal study
Ewa Janosz [6]	2 months	Case-control	2021	Germany	The European region.	Animal study
Rejean Liqun Wang [7]	4 months	Clinical trial	2009	USA	Region of Americas.	Animal study
Julia G. Payne [3]	52 weeks	Clinical trial	2016	USA	Region of Americas	Animal study
Christine L. Halbert [8]	13 months	Clinical trial	2010	USA	Region of Americas	Animal study
Mark L. Brantly [9]	1 year	Cohort	2009	USA	Region of Americas	Human study; 5 males, 4 females
Terence R. Flotte [10]	3-12 months	Cohort	2011	USA	Region of Americas	Human study; 7 females, 2 male
Terence R. Flotte [11]	1-year	Phase 1 clinical trial	2004	USA	Region of Americas	Human study; 12-20 sample size
Maria J. Chiuchiolo [12]	12 months, with a follow-up of 15 years	Cohort	2014	USA	Region of Americas	Human study
Roberto Calcedo [13]	1.5-year	Cohort	2017	USA	Region of Americas	Animal study
Mark L. Brantly [14]	180 days with a 15-year follow-up	Cohort	2006	USA	Region of Americas	Human study; 10 males, 2 female
Bishnu P. De [15]	24 weeks	Clinical trial	2005	USA	Region of Americas	Animal study
Bishnu De [16]	40 weeks	Clinical trial	2004	USA	Region of Americas	Animal study
D. Zhang [17]	7 days	Clinical trial	2003	USA	Region of Americas	Animal study

Relationship Between Gene Therapy and the Outcome of AAT Production in AAT Deficiency

Gene therapy aims to treat diseases by altering gene expression or modifying cells. Gene therapy for AATD uses recombinant viral vectors like rAAV, retroviral, lentiviral, and adenoviral vectors [18]. The first human DNA modification attempt was in 1980, but the first successful gene transfer was in 1989. In 1990, French Anderson performed the first therapeutic gene transfer. Between 1989 and 2018, over 2,900 clinical trials were conducted, mostly in phase I. Medicine became the first approved gene therapy in 2003, followed by more approvals since then [19,20]. Researchers are using the adeno-associated virus (AAV) vector for AAT deficiency, and a significant result was found (Table 3). In Table 3, sample size, what kind of vector was used, outcome, and p-value are all included.

**Table 3:** Role of gene therapy for AAT deficiency-associated lung disease. AAT: alpha-1 antitrypsin; rAAV: recombinant adeno-associated virus; SIN: Self-Inactivating Vectors; AAV: Adeno-Associated Virus; Cbx: Chromobox; EFS: Event-Free Survival; GFP: Green fluorescent protein; EF1α: Elongation factor 1α; GC: Gas chromatography; CASI: Cognitive Abilities Screening Instrument; hAAT: Highly active antiretroviral therapy; CB: Chicken Beta-Actin promoter; AE: Adverse Event

Author and publication year	Sample size (n)	Vector	Dose	Duration of therapy	Route of administration	P-value	Outcomes of gene therapy	Time for immune response	Side effects of the therapy
Marina Zieger et al. 2022 [2]	SERPINA1-e knockout mice at the ages of 18, 28, and 52 weeks (n=30)	rAAV serotype eight vectors	Two different doses (1.4 10^11 and 5.0 10^10 genome copies [gc]/mouse	36 weeks	Injection	P< 0.05	rAAV-AAT -significantly improved lung function - preserve the elastic properties of lung tissue and the integrity of alveolar walls -can compensate for the loss-of-function disease -restore protease-antiprotease balance - prevent development of or slow down the progression of SERPINA1-associated lung disease	36 weeks	N/A
Ewa Janosz et al. 2021 [6]	B6;129P2-Csf2rb2tm1Mur (Csf2rb–/–) mice at the age of 15–21 weeks (n=12)	SIN-lentiviral	1.58 ± 0.67 × 10^8 for Cbx-EFS-GFP, 1.62 ± 1.13 × 10^8 for Cbx-EFS-AAT, 2.03 ± 2.2 × 10^8 for Cbx-EF1α-AAT, and 0.4 ± 0.3 × 10^8 for CAG-AAT.	2 months	Inhalation	P< 0.01	- AAT can be expressed and secreted	Two Months	N/A
Liqun Wang et al. 2009 [7]	Eight- to ten-week-old C57/Bl6 mice (n= 100)	AAV vector	30-μl aliquot (1 × 1010 vg) of virus	4 months	Intranasal, Intratracheal	P< 0.05	AAV8 vectors -resulted in the most robust AAT expression more in tracheal administration than intranasal	4 weeks	N/A
Julia G Payne et al. 2016 [3]	Eight-week-old female C57BL/6J mice (n=35)	Recombinant AAV8 vectors	1 × 1011 GC of AAV8-CASI-hAAT (n = 20) or control vector (AAV8-CASI-GFP, n = 15)	52 weeks	IT, intramuscular or intraperitoneal injections	P < 0.0001	AAV8-mediated hAAT expression -diminished the degree of lung injury	8 weeks	N/A
Christine L Halbert et al. 2010 [8]	Dog, Mouse (n=12)	recombinant adenovirus AAV6	Mice: 0.5 or 1.0 × 10e11 Dogs: 2 × 10^13 vg	13 months	nasal	Significant	AAV6 vector encoding hAAT -for mice hAAT protein was detected in alveolar cells, epithelial cells of small and large airways -for dogs, hAAT proteins were detected alveolar and distal lung	1-2 months	N/A
Mark L Brantly et al. 2009 [9]	Human five female, four male (n=9)	recombinant adenovirus	6.9 × 10e12, 2.2 × 10e13, 6.0 × 10e13 vg	1 year	intramuscular	P <0.05	rAAV administration intramuscularly showed that despite the immune response, some of the transduced cells survive and continue to express the therapeutic protein (AAT)	90 days	Bacterial epididymitis, mild bruising, swelling, redness in the injection site
Terence R Flotte et al. 2016 [10]	Human, 7 Female, 2 Male (n=9)	recombinant adenovirus	6.0×10(11), 1.9×10(12), and 6.0×10(12) vector genomes/kg	3-12 months	intramuscular	Significant	Results from this clinical trial support the feasibility and safety of AAV gene therapy of AAT deficiency and produce AAT at the therapeutic level	90 days	injection site reactions (discomfort, erythema, bruising, or pain) Mild and transient discomfort at the injection sites
Terence R Flotte et al. 2004 [11]	Human (n=12-20)	recombinant adenovirus	(rAAV-CB-hAAT) dose levels (2.1 3 1012, 7.0 3 1012, 2.1 3 1013, and 7.0 3 1013 vg).	365 days	Intramuscular				Nausea, diarrhea, Headache
Maria J Chiuchiolo et al. 2014 [12]	Human (n=20)	recombinant adenovirus	8* 1012- 8* 1013 gc	365 days with a 15-year follow-up	intrapleural and intravenous	Significant	persistence of AAV vectors results in sustained transgene expression		pain, discomfort, bleeding at the site of needle insertion, chest pain
Roberto Calcedo et al. 2016 [13]	Mice (n=9)	AAV	6e11, 1.9e12, and 6e12 viral ge- nomes/kg,	1.5 years	Intramuscular	Significant	AAV1 capsid and AAT transgene immunity -developed AAV1 capsid-specific T cells -two subjects developed AAT transgene-specific T cells		N/A
Mark L Brantly et al. 2006 [14]	Human, 10 male, two female (n=12)	rAAV2 AAT	2.1 1012 vector genomes (VG) to 6.9 1013 VG	180 days with a 15 years follow-up	intramuscular	Significant	rAAV2 AAT - serum AAT levels were high at baseline	90 days	All potentially related AEs were minor
Bishnu De et al. 2004 [15]	Mice (n=6)	AAV serotype 5, AAV5CUha1AT, AAVrh.10A1AT	AAV5CUha1AT and AAV2CUha1AT vectors (3 ×10^10 genome copies)	168 days/ 24 weeks	intrapleural and intramuscular routes	significant	AAVrh.10 - most effective known AAV vector for intrapleural gene delivery and has the advantage of circumventing human immunity to AAV	4 weeks	N/A
Bishnu P De et al. 2006 [16]	Mice (n=25)	AAV5-based vector	AAV5CUha1AT and AAV2CUha1AT vectors (3 1010 genome copies)	40 weeks	Intrapleural or Intramuscular	Significant	AAV5- CUha1AT vector delivery by intrapleural administration results in approximately 10-fold higher levels of serum a1AT	4 weeks	N/A
D Zhang et al. 2003 [17]	Female mice (n=10)	adeno-associated virus (AAV)	5×10^5 or 1×10^6 of the gene-transferred J774A.1 macrophages in a volume of 50 ml	7 days	Intratracheal	P<0.01	AAV -significantly and persistently elevated hA1AT within the lung -This gene transfer using transfected macrophages may be useful as a therapeutic approach		N/A

In 2016, Julia G Payne et al. demonstrated that in vivo gene delivery is promising but challenging for treating monogenic diseases like AATD. Researchers have developed Adeno-associated virus vectors** **with different specificities to target multiple organs. The AAV8-CASI-luc vector delivered intratracheally (IT) achieved sustained lung transgene expression for at least 52 weeks, with unexpected bioluminescence in the abdomen, indicating transduction beyond the lungs. Most abdominal bioluminescence declined after 24 weeks, but 29% of mice had persistent expression up to 72 weeks. Green fluorescent protein (GFP) + cells** **were found in the lung areas, confirming transgene expression. This method provided therapeutic AAT protein levels, reducing experimental emphysema severity in mice. Additionally, AAV2/8 transduced liver cells, suggesting potential for human gene therapy. These findings support lung-directed AAV2/8 as a promising approach for AATD gene therapy [3].

Another study also denoted that AAV gene therapy is useful for the treatment of AAT deficiency. In the study, the effectiveness of AAV mutations and serotypes for lung transduction in mice was examined. AAV8 showed the highest AAT expression in the lung, outperforming AAV9, AAV5, AAV2, and AAV2 capsid mutants. AAV9 also demonstrated better transduction and expression than AAV5. Intratracheal delivery was more efficient than intranasal delivery for most vectors. Intranasal delivery achieved 1/2 to 1/3 the serum hAAT (highly active antiretroviral therapy)** **levels of intratracheal delivery, but both methods provided long-term expression, lasting at least 4 months [7].

Bisnu De et al. showed that Intrapleural administration of AAV5-hA1AT achieved higher lung and serum A1AT levels than intramuscular delivery, with AAV5 yielding 10 times higher levels than AAV2. Major expression sites were the diaphragm, lung, and heart. At 40 weeks, AAV5-hA1AT achieved serum A1AT levels of 900 ± 50 mg/ml, surpassing the therapeutic level [15]. He again showed us in another study that after screening 25 AAV vectors, intrapleural AAVrh.10-A1AT administration resulted in 77% transgene expression in the chest area and provided long-term therapeutic A1AT levels in mice. Higher doses were needed for female mice. AAVrh.10-A1AT worked well in both preimmunized and naive mice, boosting serum levels by 300% when used after AAV5-A1AT [16].

Christine L. Halbert et al. tested lung-directed gene therapy for AATD using an AAV6 vector with human AAT (hAAT) copy DNA (cDNA)** **in mice and dogs. In normal mice, hAAT levels peaked near therapeutic levels at 1-2 months but dropped by 6 months due to immune responses. Immunodeficient mice maintained near-therapeutic hAAT levels for a year. ELISA showed higher hAAT in lung fluid than in plasma, with AAT protein found in various lung cells. In dogs, immunosuppressive drugs were used to prevent immune reactions. The AAV6 vector efficiently transduced lung cells, resulting in therapeutic AAT levels in the lungs for 58 days to 4 months. A non-immunosuppressed dog showed no serum expression after 45 days and had a lymphoproliferative response to the AAV capsid. This study highlights the potential for lung-directed AAT gene therapy and the need to prevent immune responses for sustained expression [8].

In another phase 1 trial, nine subjects (five male, four female) with AATD received intramuscular injections of a recombinant adeno-associated virus (rAAV) vector expressing normal AAT. They were given doses ranging from 6.9×10^12 to 6.0×10^13 vector genome particles. The treatment was well tolerated, with mild local reactions and one unrelated serious adverse event. Despite immune responses to the vector, sustained expression of M-specific AAT was observed at 0.1% of normal levels for at least a year. Even though T cell responses were detected, transduced cells persisted, suggesting potential for long-term gene therapy. Though expression levels were lower than desired, the study demonstrated a promising safety profile, hinting at the possibility of higher doses without requiring immune suppression [9].

Terence R. Flotte et al., in their phase 2 trial, showed that nine AAT-deficient individuals got injections of an rAAV vector carrying human AAT. Doses varied from 6.0×10^11 to 6.0×10^12 vector genomes/kg. AAT levels peaked at day 30 and stayed up for 90 days. The treatment was well tolerated, with mild injection reactions and no serious side effects. Expected immune responses to the vector, but not to AAT, were seen. Muscle biopsies showed AAT expression and mild inflammation, suggesting AAV gene therapy for AAT deficiency is safe, though improving therapeutic AAT levels is necessary [10].

AAV gene therapy for AAT deficiency expressed promising results in phases I and II trials, with stable increases in wild-type AAT levels. In a phase II clinical trial for AAV gene therapy in AAT deficiency, nine subjects received different doses of AAV1-AAT intramuscularly. Analysis showed that all subjects developed AAV1 capsid-specific T cells, but only two developed AAT transgene-specific T cells, homozygous for the z-AAT mutation, showed sustained T-cell responses to AAT peptides, peaking at 3 months and persisting for over a year. Further investigation revealed this was due to a rare HLA-C (human leukocyte antigen) allele in one patient, leading to a robust T-cell response against the AAT protein. This underscores the genetic influence on gene therapy outcomes. This highlights the importance of considering polymorphisms and HLA restrictions when designing gene therapy interventions, as they can influence immune responses to the therapeutic product [13].

Discussion

Summary of Findings

In studies exploring AAV gene therapy for AAT deficiency, researchers have made significant progress. There are 14 articles included in this systematic review that show how AAV gene therapy helps with AAT deficiency. They've developed AAV vectors with different specificities for targeting organs and achieved significant results with minimal side effects in animal and human models. Some study highlights the potential of AAV2/8 vectors for lung-directed gene delivery; hence, further improvement is needed [3,7]. Another study denoted that intrapleural AAVrh.10-A1AT injection produced long-term therapeutic A1AT levels with 77% transgenic expression in the chest region both in pre-immunized and naïve mice [16]. Other results in mice and dogs show the feasibility of expression of therapeutic levels of AAT in the lungs after AAV vector delivery and advocate for approaches to prevent cellular immune responses to AAV capsid proteins for the persistence of gene expression of the human [8]. In the phase 1 experiment, nine AAT-deficient humans were injected intramuscularly with a rAAV1-AAT vector. In the group receiving the highest dose, AAT expression persisted at subtherapeutic levels for a minimum of a year despite T-cell responses occurring within 14 days. AAT expression was sustained in one middle-dose participant and all high-dose subjects, peaking around 90 days earlier than in the non-clinical trial. There was evidence of no T cell or antibody response to the AAT transgene, indicating the possibility of long-term gene therapy [9].

However, challenges remain, including immune responses and variability in patient outcomes, highlighting the need for further research. Additionally, genetic factors, such as polymorphisms and HLA restrictions, play a significant role in gene therapy outcomes and need to be considered in treatment design [13]. Overall, AAV gene therapy holds the potential for treating AAT deficiency, but continuous studies are necessary to optimize its effectiveness and safety.

Agreement and Disagreement With Contemporary Research

Alpha-1 antitrypsin deficiency (AATD) is a genetic disorder that leads to lung and liver disease. Common treatments are similar to those for COPD and emphysema, including avoiding smoking, bronchodilators, antibiotics, corticosteroid inhalations, and beta-agonists, but these do not increase AAT levels in the serum. Augmentation therapy is the only particular treatment available for AATD. It involves weekly intravenous infusions of pure AAT protein to keep serum levels above 80 mg/dl (11 μM). This treatment did not greatly affect mortality or aid patients with severe liver illness or lung function loss, but it does help lower COPD exacerbations and slow the loss of lung density. It is costly and has short-term effects, highlighting the need for better treatments. Gene therapy for AATD, using recombinant viral vectors like rAAV, retroviral, lentiviral, and adenoviral vectors, shows promise. Initial studies with retroviral vectors demonstrated hAAT expression but had risks like insertional mutagenesis. Adenoviral vectors avoid genome integration but can cause severe immune responses and require multiple administrations. Despite these challenges, gene therapy remains a promising alternative for more effective AATD treatment [18].

A common single-gene illness, AATD is a good candidate for gene therapy, particularly in individuals with lung diseases. Plasma levels of wild-type (M) AAT must be raised to a minimum of 11 micromolar (571 mcg/ml). Given that the short AAT gene functions in plasma, it is an attractive target, and every cell that secretes AAT has therapeutic potential. Although they only reached 3% to 5% of the goal levels, clinical experiments employing viral and nonviral vectors (recombinant adenovirus, gamma retrovirus, and rAAV) have shown early promise. The European Medicines Agency suggested the first gene treatment for AATD using an intramuscular rAAV vector [21].

A 2014 article showed that intramuscular rAAV1-AAT in clinical trials achieved 3% of the target AAT levels, which is promising. Future improvements may include intrapleural and airway delivery, miRNA (microRNA) vectors, iPS cell (induced pluripotent stem cells) platforms, and genome editing [22].

Gene therapy for alpha-1 antitrypsin deficiency (AATD) has progressed from animal models to human trials using muscle-targeted adeno-associated virus (AAV) vectors [23]. Early studies began with AAV2 in 2006, but detection of gene-derived alpha-1 antitrypsin (hAAT) was limited due to residual augmentation therapy [14]. AAV1 emerged as more effective, producing 100 times higher hAAT levels in mice and showing manageable muscle inflammation without germline transmission [24]. In Phase 1 trials (2009), nine patients received AAV1, experiencing mild injection-site reactions and transient vector DNA in blood [9]. Phase 2 trials, using an improved herpes simplex virus (HSV)-based system for higher vector yield, achieved better therapeutic outcomes at increased doses [10].

AAT is inactivated by cigarette smoke, pollution, and oxidation. Researchers used an adeno-associated virus (AAV8) vector to create an oxidation-resistant version of AAT (A213/V351/L358; 8/augmented left vector). Mice received both intravenous and intrapleural administration of this variety. High, dose-dependent amounts of AAT were found in both serum and lung fluid; in contrast to normal AAT (A213/M351/M358), 8/AVL retained its protease-inhibitory activity in the face of oxidative stress. For AAT deficiency, this second-generation gene therapy provides efficient lung protection even in the face of oxidative stress [25].

Despite slow progress, advances in viral and nonviral vectors are promising. Combining liposome technology with innovative vector design may enable long-term expression of the AAT gene at therapeutic levels. While targeting the liver for AAT gene expression is logical, local expression in lung cells may be easier and offer unique therapeutic benefits. New technologies might allow direct in vivo correction of the mutant AAT gene [26].

Study limitations

Our study has some limitations. Firstly, we have a low number of included studies, with a total of 14 studies included for review, most of them being animal studies. No pediatric patients were included. We followed definite inclusion and exclusion criteria (Table 1) to avoid bias in the inclusion of studies. Moreover, we included studies that are only written in English. Also, regarding the risk of bias assessment, most of our studies had a high risk of selection bias due to the limited participants. In addition, we could not do a meta-analysis due to the heterogeneity of the included articles.

## Conclusions

Gene therapy using viral vectors has a potential role in producing AAT protein. However, its impact on mortality, treatment frequency, side effects, and immune response are still under evaluation. Human trials are necessary to establish the effectiveness and safety of gene therapy for AAT-deficiency lung disease. In conclusion, while initial studies are encouraging, more research is needed to confirm the role of gene therapy in treating AATD-mediated lung disease.
